# Reflections on the Case of a Young Girl, Who Died with Symptoms of Hydrophobia

**Published:** 1800-07

**Authors:** C. Bosquillon

**Affiliations:** Physician to the Chief Hospital, (Grand Hospice) at Paris


### *Reflections on the Case of a young Girl, who died with Symptoms of Hydrophobia.*

By C. BOSQUILLON, Physician to the Chief Hospital, (Grand Hospice) at Paris.

I HAVE proved (says the Doctor) in my Commentaries on the Elements of Practical Medicine by CULLEN, that terror alone may determine the symptoms of hydrophobia or madness, in those who had been bitten by an animal suspected to be attacked with this disorder. Numbers of persons have been seen to labour under symptoms of this nature, and to recover on being informed that the dog that had bitten them was not mad. There are others, on the contrary, have become suddenly hydrophobial drophobial on learning, at the end of a confiderable time, that the perfons bitten at the fame period with themfelves had died with fymptoms of hydrophobia. The informations which I have received on the cafe of the young perfon in queftion, have convinced me that terror alone produced all the terrible effects which fhe had experienced. She was bitten the 11th Frimaire, (Dec. 1,) by a little dog of fix months old, naturally fnappifh; it had, however, never ceafed to obey the orders of its mafters, nor did it appear to know them lefs than ufual; its eyes had nothing menacing in their look, nor was there any froth about the mouth. The family was juft informed of a perfon recently dead by madnefs; it was imagined that this little dog was attacked by the fame malady; they refolved to drown it, and this they did without affuring themfelves of the apprehended fact.

What an impreffion might not this be fuppofed to make upon a girl of fourteen years and a half, extremely fenfitive, and of a very lively imagination! From this moment fhe became deeply thoughtful; about forty days afterwards fhe hurt the finger bitten, in attempting to cleave a piece of wood; the cicatrice of the wound where fhe was bitten opened again, and her uneafinefs greatly increafed. During the periodical evacuation incidental to her fex, fhe had occafion to put her hands in cold water; a fuppreffion took place, and that was followed, the 30th Nivofe, (January 19,) fifty-one days after the bite, with a violent head-ach accompanied with fever, a contraction of the throat, a frequent fneezing, and unquenchable thirft. Fumigations were made ufe of to relieve the head-ach, but prefently univerfal tremblings and convulfions took place. She drank with perfect eafe till the 2d Pluviofe (Jan. 21), when fhe let fall a glafs of water which her mother offered her; her hand and her whole body, her countenance efpecially, were at that inftant greatly agitated by convulfive movements. Thofe about her, being greatly frightened, occafioned her to be conveyed that fame evening to the Great Hofpital. She was much affected to find they tied her in her bed, which it is the cuftom to do with hydrophobial patients. She paffed the night in a difturbed condition; the next morning fhe pufhed back a glafs of water they offered her, and fell into convulfions; fhe was, neverthelefs, very thirfty. They made her drink out of a biberon; fhe fwallowed the liquid with precipitation, and complained even that they did not pour it out faft enough. She preferred water tinctured with red, or mixed with a little fweet orange-flower water; but at every time fhe fwallowed, the convulfive movements returned: neverthelefs, fhe endured the fight of a large veffel which was brought to her for the purpofe of bleeding her in the foot. Her feet were left in it a long time, and ftill fhe did not fall into convulfions. Her imagination became greatly inflamed; fhe talked with much volubility, and bitterly complained that fhe had heard nothing but of madnefs fince fhe was bitten. I faw her the 4th Pluviofe (January 23,) an hour before her death; fhe was greatly dejected and funk down, and had a pale, wafted countenance; every thing announced that fhe was out of the reach of all relief. Hydrophobial patients, on the contrary, generally die in ftrong convulfions, which come on even frequently at the inftant when we think them better. Their colour changes but little; which, without doubt, has given reafon for the world to believe that they have been ftifled to death; moreover, the convulfions have only appeared the third day of the diforder; they have been preceded by fever, and a contraction of the throat, apparently the effect of inflammation in the œfophagus and the trachea arteria; indeed, it can hardly be doubted but that is the cafe, when we confider the extraordinary quantity of faliva, and of mucus, with which we find thefe parts filled after death, and of the rednefs throughout them. In fhort, the true hydrophobia, as well as other convulfive idiopathic diforders, is never preceded by fever; it comes on at once. All this proves therefore that terror alone threw this young girl into the frightful ftate which occafioned her death.

It was neceffary, in order to preferve her, that thofe who furrounded her fhould have taken quite a contrary courfe to that which they obferved, and have compofed and comforted her mind; and, above all, have perfuaded her that the animal was not mad. Tranquillity of the mind alone may keep off this cruel difeafe. It becomes effential then, as well for thofe who have been bitten, as for the progrefs of the art, to keep the dogs, or other animals, fuppofed mad, fhut up fafely in a convenient place for five or fix weeks; the madnefs always fhows itfelf in the courfe of this time, and they die in a few days when the hydrophobia comes on. When they are attacked by any other diforder, the proof of what it is is foon obtained. By fuch a procedure we may avoid creating a great many torments or apprehenfions, at leaft in thofe who have been bitten by fuch animals; we fhould not be obliged to keep them, for two months together, tied down in their beds, as is the common cuftom, fubjecting them to a treatment which may well augment their fears, and determine or fix that diforder which it is our defign to prevent. It is proper alfo that the public fhould know, that it is proved that in all thofe who have been bitten by animals with the hydrophobia, a twentieth part at moft experience the fymptoms of hydrophobia, and that thefe are generally perfons of minds the moft fenfitive, and fubject to to alarm. I do not know that children, who have not attained the age of reaſon, have ever experienced theſe ſymptoms.

